# Investigating Motor Competence in Association with Sedentary Behavior and Physical Activity in 7- to 11-Year-Old Children

**DOI:** 10.3390/ijerph15112470

**Published:** 2018-11-05

**Authors:** Anoek M. Adank, Dave H. H. Van Kann, Joris J. A. A. Hoeboer, Sanne I. de Vries, Stef P. J. Kremers, Steven B. Vos

**Affiliations:** 1 School of Sport Studies, Fontys University of Applied Sciences, 5644 HZ Eindhoven, The Netherlands; d.vankann@fontys.nl (D.H.H.V.K.); steven.vos@fontys.nl (S.B.V.); 2 Department of Industrial Design, Eindhoven University of Technology, 5600 MB Eindhoven, The Netherlands; 3 Department of Health Promotion, Nutrition and Translational Research Institute Maastricht (NUTRIM), Maastricht University, 6200 MD Maastricht, The Netherlands; s.kremers@maastrichtuniversity.nl; 4 The Hague University of Applied Sciences, Research group Healthy Lifestyle in a Supporting Environment, 2521 EN The Hague, The Netherlands; j.j.a.a.hoeboer@hhs.nl (J.J.A.A.H.); s.i.devries@hhs.nl (S.I.d.V.)

**Keywords:** motor competence, sedentary behavior, moderate-to-vigorous physical activity, primary schoolchildren, accelerometer

## Abstract

Children’s motor competence (MC) has declined in the past decades, while sedentary behavior (SB) has increased. This study examined the association between MC and physical activity (PA) levels among primary schoolchildren. Demographics, body height and weight, MC (Athletic Skills Track), and PA levels (ActiGraph, GT3X+) were assessed among 595 children (291 boys, mean age = 9.1 years, SD = 1.1). MC was standardized into five categories: from very low to very high. PA levels were classified into SB, light PA (LPA), and moderate-to-vigorous PA (MVPA). Mixed-model analyses were conducted with PA levels as dependent variables and MC as the independent variable, while adjusting for age, gender, and body mass index (BMI) z-score on the individual level. A negative association between MC and SB and a positive association between MC and MVPA were found. The strength of both associations increased as children expressed lower or higher levels of MC. MC is an important correlate of both SB and MVPA, particularly for children with very high or low MC. Developing and improving children’s MC may contribute to spending less time in SB and more time in MVPA, particularly for high-risk groups, i.e., children with low MC. Moreover, addressing MC development and PA promotion simultaneously might create positive feedback loops for both children’s MC and PA levels.

## 1. Introduction

Physical activity (PA) and sedentary behavior (SB) are important health determinants. Sufficient regular PA has positive effects on physical and psychological well-being [1], while excessive SB is associated with negative health outcomes [2,3]. PA and SB patterns in childhood lay the foundation for a healthy lifestyle in adulthood [4,5]. Inactive children and adolescents have a greater likelihood of becoming physically inactive adults than active children, emphasizing the importance of increasing PA and limiting SB among children. Nevertheless, children’s physical inactivity and sedentary lifestyles are still increasing worldwide [6]. Similar to children in other countries, 45% of the Dutch primary schoolchildren do not meet the World Health Organization’s recommendation of at least 60 min of moderate-to-vigorous PA (MVPA) a day [7].

Socioecological frameworks of health behavior, such as the Environmental Research framework for weight Gain prevention (EnRG framework) [8], consider PA and SB to be a result of an interaction between individual and environmental factors. From this ecological viewpoint, motor competence (MC) can be affected by environmental factors, e.g., physical education (PE) lessons, and forms an individual predictive variable for PA and SB. Studies by De Meester et al. [9] and Lubans et al. [10] support this hypothesis by showing positive associations between children’s level of MC and the time children spent in PA. MC is ideally learned and developed during childhood [11], yet declines in children’s MC have been reported over the last years [12,13].

According to the concept of physical literacy [14], physical competence (including MC) and engagement in PA are interconnected key elements for the development of active lifestyle patterns. This suggests a reciprocal relationship between MC and PA; children with high MC are more likely to participate in PA than children with low MC, and vice versa [15,16].

The impact of the relationship between MC, PA, and SB might be influenced by a set of individual demographic variables. First, children’s weight status is recognized as a strong correlate of PA and SB. Overweight and obese children have higher levels of SB [17] and lower levels of PA than children with a normal weight [18]. In addition, body mass index (BMI) and MC are negatively associated [10]. Non-overweight children perform better on locomotor skills than overweight children [19]. Gender differences have also been found in PA and SB. Girls are more sedentary than boys [17] and spend less time on MVPA than boys [20,21,22]. Age is also a confounder in the relationship between MC, MVPA, and SB. Older children spend more time in sedentary activities [23,24,25] and engage in PA for shorter times than younger children [22,26,27,28], independently of their MC.

Increased insight into the relationship between MC, PA, and SB and the confounding effects of important demographic variables (i.e., BMI, gender, and age) on this association is necessary to develop strategies that enhance healthy lifestyles in children. However, studies addressing this relationship are scarce, particularly considering daily time spent in SB.

Therefore, the purpose of this study was to investigate the association between primary schoolchildren’s MC, daily PA, and SB as well as studying the potential confounding effects of BMI, gender, and age on the relationship between MC and PA, and MC and SB, respectively.

## 2. Materials and Methods

For the current study, baseline data were taken from the SALTO study. The SALTO study focused on the relationship between PE characteristics and daily SB and PA among primary schoolchildren. It included 10 primary schools in Eindhoven and Maastricht, two cities in the southern part of the Netherlands (over 200,000 and 100,000 citizens, respectively). Schools were recruited in collaboration with two local school boards. The selection criteria were “socioeconomic status (SES) of the neighborhood in which schools are located” and “an equal number of schools with and without specialist PE teachers”. First, schools in Eindhoven with specialist PE teachers were recruited, followed by comparable schools (based on SES) without specialist PE teachers in Maastricht.

Children in grade 4 (aged 7–9 years) and grade 6 (aged 9–11 years) were invited to participate in the SALTO study. Data collection took place between May and July 2017. The children were verbally informed about the content of the SALTO study by researchers during school visits. The parents were asked to provide written informed consent for their children’s anthropometry and PA measurements. All children were invited to take part in the measurement for MC during a regular physical education (PE) lesson. The SALTO study obtained ethical approval from the Ethical Research Committee of Fontys University of Applied Sciences (reference number FCEO 24-03 Adank). 

In total, 1126 children were invited to participate in the SALTO study, of whom 728 (64.7%) provided written parental informed consent to conduct anthropometry and PA measurements. Children who did not provide complete data on all variables, e.g., due to invalid accelerometer data or absence during measurements, were excluded from the analyses.

### 2.1. Physical Activity and Sedentary Behavior

Accelerometers (ActiGraph GT3X+; 30 Hz; 10 s Epoch) were used to objectively measure daily SB and PA. Participating children were equipped with an accelerometer during classroom visits conducted by a researcher and research assistant. The researcher checked that the children wore the elastic belt with the accelerometer positioned on the right hip. Children were instructed to wear the accelerometer for seven consecutive days, except during water-related activities and when asleep. The parents received written information about the accelerometer. 

The collected data were analyzed using the ActiLife software, version 6.10.4. Evenson’s cut-off points [29] were used to categorize PA into three levels: SB (<101 counts per minute (CPM), light PA (LPA) (101–2295 CPM), and MVPA (>2295 CPM). Choi’s [30] wear time validation criteria were applied; a valid school day was defined as the provision of at least 480 min of valid wear time between 6:00 a.m. and 11:00 p.m. The first measurement day was deleted to prevent equipment reactivity [31]. Children providing at least three valid school days of PA data were included for further analyses. In the current study, weekend days were excluded.

### 2.2. Anthropometry

Children’s body height was measured using a SECA portable stadiometer (*model 213*; SECA, Germany). Body weight was measured using a calibrated portable electronic scale (*model 803*; SECA, Germany). A trained research assistant carried out these measurements in a separate section during a PE lesson. For standardization, all children wore light clothes (shorts and t-shirt) and no shoes. BMI z-scores were calculated using a Dutch reference population to standardize for age and gender [32] and were classified by weight status according to the 2007 recommendations [33], resulting in four categories (underweight (BMI z-score ≤ −1.65), healthy weight (−1.64 ≤ BMI z-score ≤ 1.03), overweight (1.04 ≤ BMI z-score ≤ 1.64), and obese (BMI z-score ≥ 1.65)).

### 2.3. Motor Competence

The Athletic Skills Track (AST) was used to assess children’s MC. MC in the current study is defined as the degree of skilled performance in a wide range of motor tasks as well as the movement coordination and control underlying a particular motor outcome [34]. The AST is a feasible, valid, and reliable assessment tool for measuring MC that can easily be incorporated in PE [35,36]. The track is an obstacle course consisting of seven fundamental motor skills tasks: balancing, bunny hopping, hopping, walking on the hands and feet, running, rolling, and clambering. The track differs in task complexity for grade-4 children (AST-2) and grade-6 children (AST-3) and should be completed as fast as possible.

The measurement took place during a PE lesson. The class was divided into three groups. A trained research assistant demonstrated and explained the AST to the group (8–10 children). After this instruction, the children were asked to practice running the track three times. During this try-out, the children received feedback from the research assistant as necessary. The children then individually performed the track as quickly as possible, while the research assistant measured the time with a stopwatch. The individual raw completion scores (time in 0.1 s) were recorded. Time scores were standardized, conforming to the age- and gender-specific AST norm scores, ranging from 1 (MC far below average) to 5 (MC way above average) [36].

### 2.4. Procedure

Involved researchers (*N* = 3), research assistants (*N* = 2), and PE teachers (*N* = 7) were trained to assess an MC test during an afternoon session lasting 3 h by two developers of the AST and received instructions on the measurement protocol for anthropometry and PA assessment. Gender and age (date of birth) were received for all children with parental consent.

### 2.5. Statistical Analyses

Data were analyzed using SPSS Statistics, version 24 (IBM Corp., Armonk, NY, USA). PA levels were converted into daily share of time spent in SB, LPA, and MVPA during school days. Share of time spent in SB, LPA, and MVPA during school days was the dependent variable. Standardized MC scores, gender, age, and BMI *z*-scores were considered independent variables. For all analyses, the statistical significance was set at *p* < 0.05.

Descriptive statistics were calculated for demographic variables, MC, SB, LPA, and MVPA outcomes. Independent samples *t*-tests were used to compare the outcomes between boys and girls, between grade-4 and grade-6 children, and between non-overweight and overweight children. Mixed-model analyses were conducted to understand the association between the independent variables and PA levels (SB, LPA, and MVPA). School was specified as a random intercept to correct for a nested structure of the data within schools. On a second level, individual characteristics were included to examine the association between MC on PA levels while correcting for age, gender, and BMI z-scores. The standardized MC scores were recoded into dummy variables. The baseline category was MC3 (normal level of MC). 

## 3. Results

In total, 595 children (mean age = 9.1, SD = 1.1) provided complete data on all variables. The sample was equally distributed among boys (*N* = 291) and girls (*N* = 304). There was a slight overrepresentation of grade-6 children (*N* = 307/52%).

The mean score for MC was 3.18 (SD = 1.01, range 1–5). A total of 17% (*N* = 103) of the sample scored below average (score 1 or 2), and 30% (*N* = 178) scored above average (score 4 or 5). No gender difference was found for MC. The mean score was significantly higher in grade-4 children than in grade 6-children. Overweight children scored significantly lower on MC than non-overweight children (Table 1).

For BMI z-scores, there were no significant gender differences nor differences between grade-4 and grade 6-children found (Table 1). Based on BMI *z*-scores, 6.9% of the children were underweight, 8.1% were overweight, and 3.2% were obese.

The mean accelerometer wear time on a school day was 766 min (SD = 64.7). Children were sedentary for 480 min (SD = 66.6) on a school day. They spent 228 min (SD = 40.7) of the time in LPA and 58 min (SD = 21.0) in MVPA. Gender differences were found for SB as well as for LPA and MVPA. Girls spent significantly more minutes a day in SB than boys (492 min vs. 467 min) and fewer minutes in LPA (224 vs. 233 min) and MVPA than boys (50 min vs. 67 min). Children in grade 4 showed significantly lower levels of SB than grade-6 children (456 min vs. 502 min). Children in grade 4 spent significantly more time in LPA (238 vs. 220 min) and MVPA than grade-6 children (59 vs. 57 min). Overweight children spent significantly less time in MVPA compared to non-overweight children (Table 1).

Mixed-model analyses were conducted for SB, LPA, and MVPA separately. The best fitted models for SB, LPA, and MVPA included main effects of MC as well as gender, age, and BMI z-scores (Table 2).

**Figure 1:**
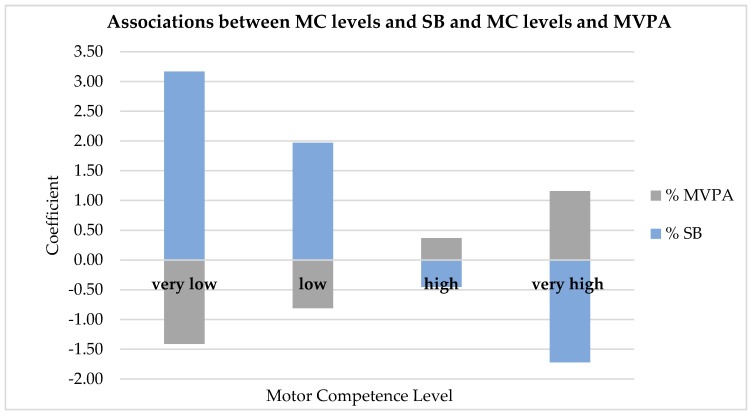
Associations (coefficients) between MC categories (MC1 = very low; MC2 = low; MC4 = high, MC5 = very high. Referent is MC3 = average MC) and % time spent in SB and between MC and % time spent in MVPA.

Regarding SB, children who scored below average (score 1 or 2) spent significantly more time in SB than children with an average score (score 3). Moreover, children with an extremely high score on MC (score 5) spent significantly less time in SB than those who had a normal score (score 3). Age (older) and gender (girl) also significantly predicted time spent in SB. The strength of the association between MC and SB increased when children expressed higher and lower MC (scores 5 and 1). The least competent children spent 3.17% (24.3 min per day) more time in SB per day compared to children with an average MC score. The most competent children spent 1.72% (13.2 min per day) less time in SB per day than children in the average MC category. For MVPA, the opposite linear association was found; compared to referent children (MC3), the least competent children spent 1.41% (10.8 min per day) less time in MVPA, whereas the most competent children spent 1.16% (8.9 min per day) more time in MVPA (Figure 1). 

With respect to LPA, children with very low MC (score 1) spent significantly less time in LPA than children with an average level of MC (score 3). There was a significant negative association between age and LPA. Boys spent more time in LPA than girls. The higher the BMI z-scores of children, the more time children spent in LPA.

Children with low MC (score 1 or 2) spent less time in MVPA than other children. Compared with children who had average MC, children with very high MC spent significantly more time in MVPA. Boys spent more time in MVPA than girls during a school day.

## 4. Discussion

This study examined the associations between motor competence, sedentary behavior, and physical activity. In line with previous studies [10,37,38,39,40], MC was positively associated with time children spent in MVPA. Interestingly, our results revealed an even stronger negative association between MC and SB. 

Although studies on the relationship between MC and SB are scarce, existing studies found similar patterns to those in the current study [41,42,43,44,45], underlining the importance of SB in relation to current declines in children’s MC. The impact of MC on SB and MPVA varied across levels of MC (Figure 1). Higher or lower levels of MC had stronger impacts on SB and MVPA in comparison with normal levels of MC. The association between MC and SB was stronger than the association between MC and MVPA.

Our findings indicate the need to focus on children’s MC as a factor that influences not only time children spent in MVPA but also sedentary time. It suggests that improvement in children’s MC, especially for those who have low MC, could be a promising strategy in reducing SB and increasing the time children spend in MVPA. The cross-sectional design limited the opportunity to test causality in the relationship between MC and SB and MVPA, respectively. Nevertheless, a reciprocal association between MC and PA levels could be hypothesized [12,14]. Reducing time spent in SB and increasing time spent in MVPA could positively affect children’s MC [14], which offers a new opportunity to decrease the negative trend in children’s MC [12,46]. On the contrary, increasing MC has the potential to decrease levels of SB and increase levels of PA. According to a systemic view, it is recommended to focus on both behaviors simultaneously to optimize conditions in which positive feedback loops can result in sustainable behavioral improvements. Yet, school-based PA programs proved that it is challenging to reach sustainable effects on PA [47], so it would be better to focus on increasing MC to initiate a positive feedback loop [15,16], e.g., through high-quality physical education lessons. Alongside developing MC, PA promotion and SB reduction remain crucial to initiate the positive feedback loops. 

There were, in line with previous studies [23,27,48], quite strong significant gender differences in sedentary time and time spent in LPA and MVPA. Girls spent more time in SB than boys, and boys engaged longer in LPA and MVPA than girls. This finding stresses the need to develop effective interventions specifically targeting girls’ PA levels.

In line with other studies [22,26,27,28,49,50], which showed that older children are less active than younger children, we found significant differences in time spent in LPA and MVPA between grade-4 and grade-6 children. Moreover, like other studies [23,24,25], we found a positive correlation between age and SB. In general, older children go to bed at a later time, and this extended duration of waking hours might be responsible for this finding, i.e., more television watching, computer gaming, and using social media.

In contrast to studies by Da Costa et al. and Keane et al. [17,18], we found no significant relationship between BMI and SB and between BMI and MVPA. It is possible that BMI as a predictor for SB and MVPA changes with age. Perhaps BMI differences between children and their impact on SB and MVPA become stronger and more apparent in adolescence. Our relatively small sample size of overweight children (*N* = 67 children; 11.3%) could also have impacted the lack of significant association in the current study.

Furthermore, children spent on average 8 h (of 11.5 h wear time) in SB on a school day, which is comparable with other studies [7,51,52]. These extremely high levels of SB during school days may be largely affected by the current form of school curricula in which children are obliged to sit most of the time. More active alternatives in curricula, such as integrating PA in regular educational activities [53,54] and providing less sedentary physical classroom environments [53,55] could be beneficial to decreasing sedentary time at school and subsequently affect MC positively. 

Besides strengths like sample size and objective measures, the current study is also subject to some limitations. First, this study used MC as an independent variable. Most other studies focused specifically on locomotor skills, object control skills, and stability skills as a measurement of fundamental motor skills, rather than the comprehensive measure of MC. Second, participating schools were located in neighborhoods with an average or high SES. The neighborhood SES could be a confounding variable as children raised in high SES families might have been slightly over-represented in the current study compared to average levels in the Netherlands. This may have affected the generalizability of the present study results. Yet, MC outcomes were standardized based on a national representative sample and therefore did not affect the association between MC and PA levels. Moreover, sensitivity analyses (data not shown) revealed no statistical differences in MC, i.e., selection bias, between participating and nonparticipating children in the SALTO study. Third, the outcome of SB and PA could be biased by seasonality. Children spend more time sedentary and less time in MVPA in winter [56]. Our data collection took place during the summer. Furthermore, this study focused only on school days. Further investigation of PA behavioral patterns on weekend days is recommended [57].

## 5. Conclusions

This study demonstrates a positive association between MC and MVPA and an even stronger association between MC and SB in children aged 7–11 years. Although reciprocal associations between MC and PA levels are still unclear, MC should receive more attention as a potential, crucial factor to counter the alarming declines in PA and increase of SB among children. We emphasize the prominent role physical education in primary schools should play in developing and improving children’s MC. In line with the reciprocal view, PA-supportive and SB-inhibiting activities are needed in addition to MC development to optimize PA development in children.

## Figures and Tables

**Table 1:** Descriptive characteristics (mean and standard deviation); total and stratified by gender and grade.

Schoolday										
	Total Mean (SD) *N* = 595	Boys Mean (SD) *N* = 291	Girls Mean (SD) *N* = 304	Boys–Girls *p* (t)	Grade 4 Mean (SD) *N* = 288	Grade 6 Mean (SD) *N* = 307	Grade 4-Grade 6 *p* (t)	Non-Overweight Mean (SD) *N* = 528	Overweight Mean (SD) *N* = 67	Non-Overweight-Overweight *p* (t)
Age	9.1 (1.1)	9.1 (1.1)	9.2 (1.1)	ns	8.1 (0.4)	10.1 (0.5)	<0.01 (−57.54)	9.1 (1.1)	9.1 (1.1)	ns
MC	3.18 (1.01)	3.25 (1.07)	3.12 (0.95)	ns	3.49 (0.92)	2.90 (1.01)	<0.01 (7.36)	3.23 (1.0)	2.85 (0.9)	<0.01 (2.88)
BMI (*z*-score) *	−0.22 (0.96)	−0.27 (1.01)	−0.17 (0.92)	ns	−0.19 (0.92)	−0.24 (1.00)	ns	−0.44 (0.77)	1.52 (0.44)	<0.01 (−30.71)
Wear time	766.4 (64.7)	766.6 (64.3)	766.2 (65.3)	ns	752.5 (62.8)	779.5 (63.9)	<0.01 (−5.19)	767.4 (64.2)	758.3 (68.6)	ns
% SB	62.6 (6.4)	60.9 (6.7)	64.2 (5.6)	<0.01 (6.56)	60.5 (6.2)	64.5 (6.0)	<0.01 (−7.83)	62.6 (6.2)	62.6 (7.8)	ns
% LPA	29.8 (4.9)	30.4 (5.1)	29.2 (4.6)	<0.01 (−3.04)	31.6 (4.5)	28.2 (4.6)	<0.01 (9.25)	29.8 (4.7)	30.4 (5.9)	ns
% MVPA	7.6 (2.7)	8.7 (2.8)	6.6 (2.1)	<0.01 (−10.57)	7.9 (2.7)	7.4 (2.7)	<0.05 (2.19)	7.7 (2.7)	7.0 (2.6)	<0.05 (2.11)

* Compared with the Fifth Dutch growth study [32]. MC = motor competence; wear time duration is expressed in minutes; BMI = Body Mass Index; SB = sedentary behavior; LPA = light physical activity; MVPA = moderate-to-vigorous physical activity; ns = not significant at p < 0.05.

**Table 2:** Associations between MC, BMI *z*-score, age, gender, and percentage of time spent in SB, LPA and MVPA on an average school day.

Data (*N* = 622)	% SB		% LPA		% MVPA	
	Beta (95% CI)	*p*	Beta (95% CI)	*p*	Beta (95% CI)	*p*
Age	1.46 (1.02–1.90)	**<0.01**	−1.42 (−1.76–−1.08)	**<0.01**	−0.04 (−0.23–0.14)	0.64
BMI-*z* score	−0.26 (−0.73–0.21)	0.28	0.37 (−0.01–0.74)	**<0.05**	−0.11 (−0.31–0.08)	0.26
Gender (ref = female)	−3.25 (−4.15–−2.35)	**<0.01**	1.19 (0.49–1.89)	**<0.01**	2.06 (1.69–2.44)	**<0.01**
**Motor Competence (ref = average)**						
Very low	3.17 (1.28–5.05)	**<0.01**	−1.75 (−3.21–−0.29)	**<0.05**	−1.41 (−2.19–−0.63)	**<0.01**
Low	1.97 (0.44–3.49)	**<0.05**	−1.15 (−2.34–0.03)	0.06	−0.81 (−1.44–−0.18)	**<0.05**
High	−0.45 (−1.71–0.81)	0.48	0.08 (−.90–1.06)	0.87	0.37 (−0.16–0.89)	0.17
Very high	−1.72 (−3.18–−0.27)	**<0.05**	0.56 (−0.57–1.69)	0.33	1.16 (0.56–1.76)	**<0.01**

Ref = referent; SB = sedentary behavior; LPA = light physical activity; MVPA = moderate-to-vigorous physical activity; FMS = fundamental movement skills; BMI = Body Mass Index. **Bold** = significant association.
